# Characterization of Tissue Immunity Defense Factors of the Lip in Primary Dentition Children with Bilateral Cleft Lip Palate

**DOI:** 10.3390/jpm14090965

**Published:** 2024-09-11

**Authors:** Laura Ozola, Mara Pilmane

**Affiliations:** 1 Institute of Anatomy and Anthropology, Riga Stradins University, Kronvalda Boulevard 9, LV-1010 Riga, Latvia; 2 Children’s Clinical University Hospital, Vienības Gatve 45, LV-1004 Riga, Latvia

**Keywords:** cleft lip and palate, tissue defense factors, primary dentition, HBD, IL, Gal-10, CD-163

## Abstract

Background: Bilateral cleft lip palate is a severe congenital birth defect of the mouth and face. Immunity factors modulate immune response, inflammation, and healing; therefore, they are vital in the assessment of the immunological status of the patient. The aim of this study is to assess the distribution of Gal-10, CD-163, IL-4, IL-6, IL-10, HBD-2, HBD-3, and HBD-4 in tissue of the bilateral cleft lip palate in primary dentition children. Methods: Five patients underwent cheiloplasty surgery, where five tissue samples of lip were obtained. Immunohistochemical staining, semi-quantitative evaluation, and non-parametric statistical analysis were used. Results: A statistically significant increase in HBD-2, HBD-3, and HBD-4 was found in skin and mucosal epithelium, hair follicles, and blood vessels. A notable increase was also noted in IL-4, IL-6, and IL-10 in the mucosal epithelium and CD163 in blood vessels. The connective tissue of patients presented with a statistically significant decrease in Gal-10, IL-10, and HBD-3. Spearman’s rank correlation revealed multiple significant positive and negative correlations between the factors. Conclusions: Upregulation of CD163 points to increased angiogenesis but the increase in IL-4 and IL-10 as well as the decrease in Gal-10 points to suppression of excessive inflammatory damage. Decreased connective tissue healing and excessive scarring are suggested by the decrease in HBD-3 and IL-10 and the increase in IL-6.

## 1. Introduction

Cleft lip is a type of orofacial cleft defect that affects the upper lip and occasionally the nasal region. It can be unilateral, bilateral, or midline and complete or incomplete [1]. It is two times more common in males than in females and most often affects the left side of the face [1]. Clinically, children with cleft lip have impaired functions of speech, nutrition, and breathing, which significantly decreases the quality of life for the child and for the parents as well as creates a necessity for regular speech therapy, orthodontist, otolaryngology, and nutritionist appointments. 

In order to regain symmetry, normal function, and physiological homeostasis of the lip and nasal region, children aged 3 to 4 months undergo surgical correction of the defect [1]. The surgical correction operation is called cheiloplasty, and it is the first of many plastic surgeries that these patients have to undergo in the following years [2]. The first operation focuses on lip repair, and sometimes may include nasoalveolar molding and lip adhesion [2]. Afterward, the surgical correction is followed by definitive cheiloplasty, primary rhinoplasty, palatoplasty, and even orthognathic surgery, all performed when the patient reaches the appropriate age and indications [2].

Afterward, the children are prescribed a strict diet plan in order to limit excessive scarring and possible injury or infection to the site of the operation as well as not to disrupt the remodeling of the tissue [1]; however, due to the constant growth and evolving of the child’s orofacial region during this age, asymmetries often occur and a follow-up surgery is necessary to correct the asymmetry [1].

It is also well-known that all orofacial defects have the characteristic feature of chronic inflammation and decreased tissue healing and remodeling potential [3]. However, the fact that cleft lip has a straight connection between the inner and outer environment predisposes the idea that the inflammatory and immune processes in the cleft lip should be much more prominent than in the cleft palate, especially if the cleft lip is bilateral. 

Various cells of the immune system produce different proteins for homeostasis maintenance and protection of the tissues, such as anti-inflammatory and regulatory cytokines (interleukin-4, interleukin-10, and interleukin-6) and tissue protective factors (galectin-10 and human beta defensin-2, -3, and -4), complemented by anti-inflammatory M2 macrophages (CD163). The functions of these protective molecules are versatile and, therefore, vital in pathological conditions of the tissues.

Galectin-10 (Gal-10) is a protein that belongs to the prototype class of the galectin family of proteins and contains a specific amino acid sequence that forms the carbohydrate recognition domain [4,5]. Galectin-10 is mainly found in human eosinophils where it is one of the most abundant proteins; however, some traces of it have also been observed in other immune cells like neutrophils, basophils, macrophages, and T cells [5,6]. The functions of galectin-10 are determined by their specific location in eosinophils–immune cells that mainly carry out immune responses targeted to parasites or allergic substances [5,6]. One of the most prominent and important functions of galectin-10 in the immune system is the suppression of T-cell proliferation and receptor activation [7,8]. Therefore, a decrease in Gal-10 can oftentimes increase the count and functions of different subpopulations of T-cells [9]. It has also been noted that increased levels of galectin-10 in pregnancy-related diseases could be connected to the increased development and prolonged maintenance of chronic inflammation via different mutually correlating cytokines and chemokines [5].

CD163 is a distinct hemoglobin scavenger receptor that is expressed by macrophages and monocytes [10]. It is characterized as an important marker for M2 anti-inflammatory macrophages and their activation due to its high specificity to this cell type [11]. Expression of CD163 is induced by IL-6 and IL-10 but decreased by IL-4, TNF-α, IFN-γ, and LPS [11,12,13]. The main functions of CD163 and M2 macrophages include the secretion of IL-10, NO, and IL-6, improved wound healing and angiogenesis, inhibition of T cells, downregulation of inflammation, and even inhibition of pathogen growth via limited pathogen access to Hb and Fe [12,14].

Interleukin-4 (IL-4) is an inflammatory cytokine that regulates and promotes different inflammatory processes [15]. The main IL-4 secreting cells are T-helper-2 cells (Th2) of the immune system; however, it can also be secreted by basophils, mast cells, T follicular helper cells (Tfh), invariant natural killer T cells (iNKTs), and by eosinophils during allergic reactions or parasitic infections [15,16]. In severe inflammatory processes, IL-4 has multiple functions, mainly focusing on the promotion of tissue repair, reduction in inflammation, and maintenance of tissue homeostasis [17]. Overall, IL-4 has a protective role in tissues to prevent excessive damage caused by the organism’s immune response [18]. 

Interleukin-6 (IL-6) is a pro-inflammatory cytokine that initiates and controls the immune response, inflammatory processes, hematopoiesis, and tissue homeostasis by regulating cytokine production, acute phase protein (ACP) synthesis, cell development, growth, differentiation, activation, and adhesion [19,20,21,22,23,24,25]. It is mainly secreted by tissue macrophages and monocytes as a defense mechanism to tissue infections and injuries [23,24,25,26]. Excretion of IL-6 has also been observed when cells like fibroblasts, endotheliocytes, epitheliocytes, neutrophils, and lymphocytes come into contact with lipopolysaccharides or inflammatory cytokines, especially IL-1 and TNF-α [24,25,26,27]. IL-6 demonstrates both pro- and anti-inflammatory actions and is an important part of the immune response that connects innate and adaptive immune responses and ensures adequate functions of epithelial and mucosal barriers [20,25].

Interleukin-10 (IL-10) is one of the IL-10 family cytokines that has an anti-inflammatory effect and that ensures the suppression of excessive inflammatory responses [28]. It is secreted by the cells of the immune system—T cells (Th1, Th2, Th9, Th17, and Treg), B cells, macrophages, dendritic cells, NK cells, and even CD8+ T cells, eosinophils, and mast cells [28,29,30,31,32,33]. The most important producer of IL-10 is CD4+ T cells [34]; however, the ability to express IL-10 has also been observed in epithelial cells [28]. IL-10 is also fundamental in the process of healing and wound repair due to the fact that it increases epitheliocyte proliferation, hyaluronan synthesis, re-vascularization, and re-epithelialization [30,35,36].

Human beta-defensins are positively charged antimicrobial peptides that are characterized by their β-sheet structure, expression in the epithelial cells (mainly, keratinocytes), and antimicrobial properties [37,38,39]. All HBDs except HBD-1 are secreted as a response to inflammation or infection. The main functions of these molecules are the elimination of pathogens, improvement of wound healing, the regulation of inflammation—by controlling cell proliferation, migration, chemotaxis and cytokine production [40,41,42,43]. Therefore, they are categorized as the first line of defense of epithelial tissues that ensure immune responses and homeostasis [38,39,44].

Upon release in epithelial tissues, HBD-2 stimulates keratinocyte proliferation and migration, the chemotaxis of macrophages, attraction of T cells and dendritic cells, as well as antimicrobial effects [43,45,46,47]. Stimulation of cell activity and immune response also ensures wound healing and the epithelial barrier maintenance effects of HBD-2 [43].

The main functions of HBD-3 are the elimination of microbes, stimulation of mast cell degranulation and NK cell activity, increase in chemotaxis and vascular permeability, as well as the stimulation of keratinocyte migration, differentiation, proliferation, and production of IL-6, IL-10, and IFN-γ [41,48,49,50]. HBD-3 functions not only against Gram-negative bacteria but—unlike other beta-defensins—also against Gram-positive bacteria, therefore ensuring a full spectrum of antibacterial defense [51]. It has also been noted that HBD-3 can prevent the formation of biofilms, especially around various types of implants that have been placed in the body [52]. Overexpression of HBD-3 has been previously linked to increased wound healing and a decreased prevalence of wound infection [41].

The functions of HBD-4 include the stimulation of IL-6, IL-10, IFN-γ, and prostaglandin D_2_ production as well as mast cell degranulation and chemotaxis [39,40]. When acting upon bacteria, the antibacterial effect of HBD-4 is carried out by the formation of pores in the cell membrane of these organisms, which is negatively charged [37].

In summary, the main functions and properties of the described cytokines and factors are as follows: IL-6 is a regulatory cytokine of other cytokines; IL-4 is a dual anti- and pro-inflammatory cytokine; IL-10 is the strongest anti-inflammatory cytokine; M2 are anti-inflammatory macrophages; HBDs are protective molecules of the tissues; and the role of Gal-10 is still unclear, although its functions are mainly expressed in eosinophilic inflammation. Little is known about the correlations and links between these molecules in different pathological states of the tissues. 

Due to the fact that local tissue defense factors are crucial mediators of inflammation, the immune response, tissue healing, and remodeling, they have been previously researched in various other studies trying to connect them with the pathological characteristics of certain conditions, including pathologies of the oral and maxillofacial region like nasal polyps, periodontitis, and various types of clefts.

The aim of this study is to assess the distribution of tissue defense factors and detect the local tissue defense status in the bilateral cleft lip and palate in children of primary dentition since prominent features of the BCLP are chronic inflammation and insufficient healing. The results of this study will continue to be a part of a continuous research cycle in order to find possible links and causalities between defense factors and characteristics of clefted tissue.

## 2. Materials and Methods

### 2.1. Material Characteristics of Subjects

This research was conducted in accordance with the 1975 Helsinki Declaration (as revised in 2008). This study was independently reviewed and approved by the Ethical Committee of the Riga Stradiņš University (22 May 2003; 17 January 2013; Nr. 5/28 June 2018). All parents of the patients were fully informed about the nature of this study, and they provided written informed consent for participation in the study and its publication.

### 2.2. Selection Criteria of Patient Tissue Samples

Samples were selected according to the following inclusion criteria:

- Diagnosis of Cheilognathouranoschisis bilateralis;
- Age of primary dentition;
- No other congenital diseases;
- Absence of additional pathologies that are contraindications to cheiloplasty;
- No signs of active inflammation
- Indications for bilateral cheiloplasty.
- The following exclusion criteria were also applied:
- Age during primary dentition [53];
- Presence of other congenital diseases;
- Contraindicative pathologies for plastic surgery;
- Signs of active inflammation.

### 2.3. Characteristics of Selected Patients

Four of the selected patients were male and one was female. One patient’s mother had refused to take mandatory pregnancy medication during pregnancy and another patient’s mother had a history of cleft (Table 1). All patients underwent bilateral cheiloplasty surgery.

In total, 5 patient samples of lip tissue were obtained during cheiloplasty surgery in the Cleft Lip and Palate Centre of the Institute of Stomatology of Riga Stradins University. The samples were acquired from children aged 4 to 17 months old that were diagnosed with bilateral cleft lip (Cheilognathouranoschisis bilateralis).

### 2.4. Selection Criteria of Control Tissue Samples

The inclusion criteria for the control group were as follows:

- Absence of craniofacial clefts in patient examination, anamnesis, and/or family history;
- Absence of additional pathologies, congenital abnormalities, or damage of oral cavity tissue.

In total, 5 control tissue samples were obtained from the Institute of Anatomy and Anthropology of Riga Stradins University during post-mortem necropsies. 

One of the selected control samples was male and four were female. The age of the control group samples varied from newborn to 24 weeks old. Two of the controls were affected by asphyxia of the umbilical cord, two by sudden death syndrome, and one was aborted due to the health status of the mother (Table 2).

The approval Nr. 2-PEK-4/595/2022 for the use of control group tissue was issued on 14 December 2022.

### 2.5. Routine Staining

Firstly, the obtained tissue material was fixated for 24 h using 2% formaldehyde, 0.2% picric acid, and 0.1 M phosphate buffer (pH 7.2). Secondly, the material was processed for 12 h using Tyrode’s buffer with 10% saccharose. Thirdly, embedding of the tissues in paraffin and cutting with microtome into 5–7 μm sections was performed. Lastly, the prepared lip tissue samples were stained with hematoxylin and eosin [54].

### 2.6. Immunohistochemical (IHC) Analysis

Immunohistochemical detection of the local tissue defense factor quantity in the selected lip tissue samples was performed using the standard streptavidin and biotin method [54,55].

Firstly, antibodies were diluted in antibody diluent (code-938B-05, Cell Marque^TM^, Rocklin, CA, USA).

Secondly, tissue samples were prepared for the antibodies: previously cut tissue sections were deparaffinized, washed in alcohol and water, rinsed with TRIS buffer solution (code-2017X12508, Diapath S.p.A., Martinengo, Italy) twice for 5 min, placed in a microwave with boiling EDTA buffer (code-2017X02239, Diapath S.p.A., Martinengo, Italy) for 20 min, and cooled. Then, the samples were once again washed with TRIS buffer two times for 5 min, blocked with 3% peroxide for 10 min, and washed with TRIS buffer. 

Thirdly, the antibody reaction was performed: samples were incubated with primary antibodies for 1 h, washed 3 times with TRIS buffer, exposed to HiDef Detection^TM^ reaction amplificator (code 954D-31, Cell Marque^TM^, Rocklin, CA, USA) for 10 min at room temperature, washed with TRIS buffer, incubated with a HiDef DetectionTM HRP Polymer Detector (code-954D-32, Cell Marque^TM^, Rocklin, CA, USA) for 10 min at room temperature, followed by the last wash with TRIS buffer 3 times for 5 min each.

Lastly, the samples were prepared for sealing: tissue was coated with a DAB+ chromogenic liquid DAB Substrate Kit (code 957D-60, Cell Marque^TM^, Rocklin, CA, USA) for 10 min, rinsed with running water, counterstained with hematoxylin (code-05-M06002, Mayer’s Hematoxylin, Bio Optica Milano S.p.A., Milano, Italy), dehydrated with ethanol of increasing concentrations (70°–90°), clarified with carboxylic acid and xylol, sealed with a coverslip, and marked according to the patient number and antibody used.

The information about the antibodies used is summarized in Table 3.

### 2.7. Assessment of Local Tissue Defense Factor Quantity

Light microscopy and the semi-quantitative counting method were used to assess the relative quantity of Gal-10-, CD-163-, IL-4-, IL-6-, IL-10-, HBD-2-, HBD-3-, and HBD-4-positive structures in skin and the mucosal epithelium, connective tissue and blood vessels, salivary and adipose gland ducts, and hair follicles. Evaluation of positively stained structures visible in the visual field was performed according to identifiers summarized in Table 4. Acquisition, processing, and analyses of the tissue sample pictures were performed using a Leica DC 300F digital camera (Leica Microsystems Digital Imaging, Cambridge, UK) and the Image Pro Plus program (Media Cybernetics, Inc., Rockville, MD, USA).

### 2.8. Statistical Analysis 

IBM SPSS (Statistical Package for the Social Sciences) software version 26.0 (IBM Company, Chicago, IL, USA) was used for statistical processing of the data. Statistical significance was selected at a *p*-value < 0.05 and was used for every statistical assessment of the tests and results [58]. Semi-quantitative evaluation of the local tissue defense factor quantity produced ordinal data (non-numeric and arranged in a specific and unchangeable order); therefore, descriptive statistics, analytical statistics, and non-parametric tests were used to calculate the results and their statistical significance.

#### 2.8.1. Mann–Whitney U Test

This test was used to detect if the distribution of immunity factor quantity in the patient and control group samples was equal or not, and if the difference was statistically significant [58].

#### 2.8.2. Spearman’s Rank Correlation

This test was used to detect whether there was a statistically significant rate of connection between changes in one factor that was connected to the changes in another factor [58]. The strength of the correlation between factors was interpreted using the following definition of Spearman’s rho (r_s_) values:

- A very weak correlation: r_s_ = 0.00–0.19;
- A weak correlation: r_s_ = 0.20–0.39;
- A moderate correlation: r_s_ = 0.40–0.59;
- A strong correlation: r_s_ = 0.60–0.79;
- A very strong correlation: r_s_ = 0.80–1.00 [59].

### 2.9. Flowchart

**Figure 1:**
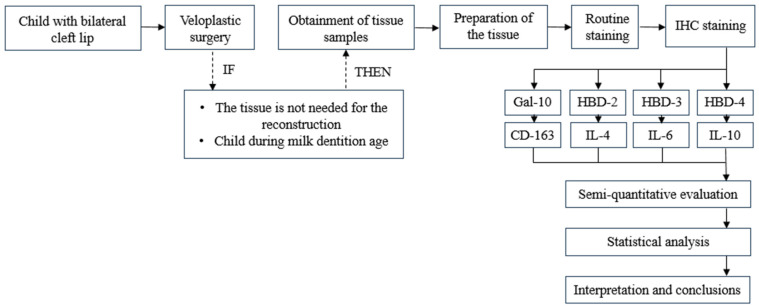
Workflow of patient tissue sample selection, processing, and research.

A visual summary of the workflow and information presented in the Materials and Methods Section is outlined in Figure 1.

## 3. Results

### 3.1. Characteristics of Routine Staining

**Figure 2:**
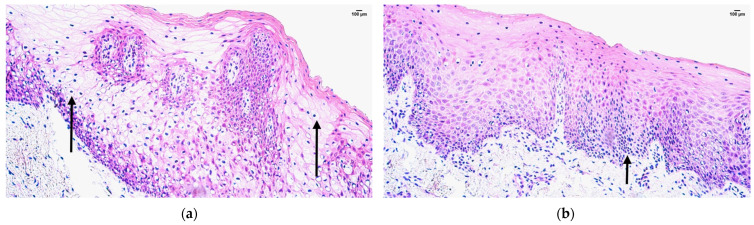
Hematoxylin and eosin routine staining of the patient tissue samples (**a,b**): note vacuolization (**a**) and inflammatory cell infiltration (**b**) in skin type epithelium of the lip (arrows). Magnification 200×.

The control sample showed features of normal lip tissue—non-keratinized stratified squamous mucosal epithelium, keratinized stratified squamous skin epithelium with sweat gland ducts and hair follicles, and underlying mucosal connective tissue with adipose glands. The patient tissue samples presented with prominent epithelial vacuolization and subepithelial infiltration of inflammatory cells (Figure 2a,b).

### 3.2. Appearance and Distribution of Gal-10

**Figure 3:**
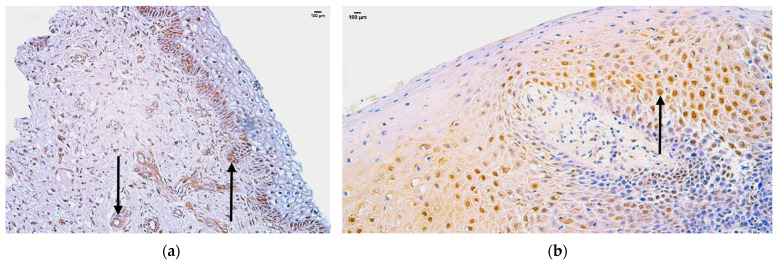
Immunohistochemistry of the Gal-10-positive structures in the control and patient tissue samples: (**a**) control sample with moderate to numerous Gal-10-positive structures in skin epithelium, and moderate in mucosal epithelium, sweat gland ducts, and connective tissue (arrows), 200×; (**b**) patient sample with few to moderate Gal-10-positive structures in blood vessels, moderate to numerous in skin epithelium, and numerous in mucosal epithelium (arrows), 200×.

In the control group, the median quantity of Gal-10-positive structures was moderate (++) in the mucosal epithelium, blood vessels, sweat gland ducts, hair follicles, and connective tissue, moderate to numerous (++/+++) in the skin epithelium, and numerous (+++) in adipose glands (Figure 3a, Table 5).

In the patient group, the median quantity of Gal-10-positive structures was few to moderate (+/++) in blood vessels, moderate (++) in sweat gland ducts and hair follicles, moderate to numerous (++/+++) in skin epithelium and adipose glands, and none in connective tissue (Figure 3b, Table 5).

Comparison of both groups using the Mann–Whitney U test illustrates a statistically significant difference (U = 0, *p* = 0.005) in the connective tissue (Table 6). In addition, there were no statistically significant differences in the skin epithelium (U = 10, *p* = 0.589), mucosal epithelium (U = 6.5, *p* = 0.368), blood vessels (U = 5.0, *p* = 0.199), sweat gland ducts (U = 2.0, *p* = 0.414), adipose glands (U = 3.5, *p* = 0.803), and hair follicles (U = 2.5, *p* = 0.195) (Table 6).

### 3.3. Appearance and Distribution of CD-163

**Figure 4:**
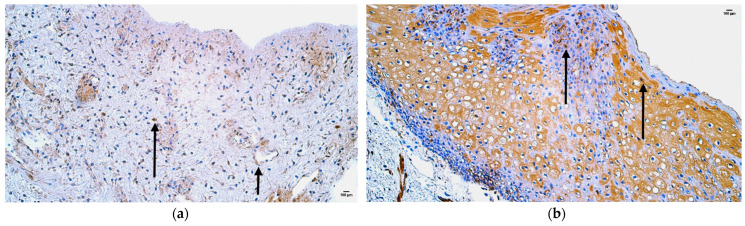
Immunohistochemistry of the CD-163-positive structures in the control and patient tissue samples: (**a**) control sample with rare occurrence of CD-163-positive structures in mucosal epithelium and sweat gland ducts, few in the blood vessels, and moderate in connective tissue (arrows), 200×; (**b**) patient sample with few to moderate CD-163-positive structures in mucosal epithelium and moderate in the skin epithelium (arrows), 200×.

In the control group, the median quantity of CD-163-positive structures was none (0) in lip skin epithelium or around adipose glands and hair follicles, rare (0/+) in the mucosal epithelium, few (+) in the blood vessels and sweat gland ducts, and moderate (++) in connective tissue (Figure 4a, Table 5).

In the patient group, the median quantity of CD-163-positive structures was rare (0/+) in the mucosal epithelium, few (+) in skin epithelium, sweat gland ducts, and hair follicles, few to moderate (+/++) in blood vessels, and moderate (++) in adipose glands and connective tissue (Figure 4b, Table 5).

Comparison of both groups using the Mann–Whitney U test illustrates a statistically significant difference (U = 3.0, *p* = 0.034) in the blood vessels. (Table 6). In addition, no statistically significant differences in skin epithelium (U = 5.0, *p* = 0.090), mucosal epithelium (U = 9.5, *p* = 0.896), sweat gland ducts (U = 3.0, *p* = 0.124), adipose glands (U =0, *p* = 0.221), and connective tissue (U = 8.0, *p* = 0.288) were found (Table 6).

### 3.4. Appearance and Distribution of IL-4

**Figure 5:**
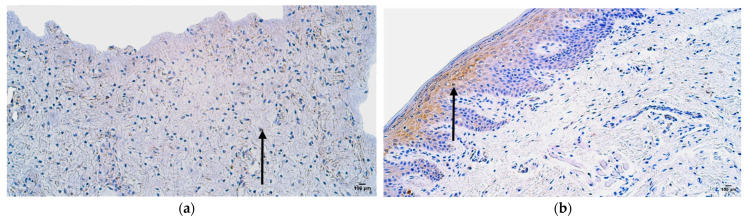
Immunohistochemistry of the IL-4-positive structures in the control and patient tissue samples: (**a**) control sample with no IL-4-positive structures in skin and mucosal epithelium and adipose glands, with rare occurrence in blood vessels, hair follicles, and connective tissue (arrows), 200×; (**b**) patient sample with few IL-4-positive structures in skin epithelium and connective tissue (arrows), 200×.

In the control group, the median quantity of IL-4-positive structures was none (0) in the skin epithelium, sweaty glands, and connective tissue, rare (0/+) in adipose glands, hair follicles, and mucosal epithelium, and few to moderate (+/++) in blood vessels (Figure 5a, Table 7).

In the patient group, the median quantity of IL-4-positive structures was none (0) in the blood vessels and connective tissue, rare (0/+) in adipose glands, few (+) in skin epithelium and hair follicles, moderate (++) in sweat gland ducts, and numerous (+++) in the mucosal epithelium (Figure 5b, Table 7).

Comparison of both groups using the Mann–Whitney U test illustrates a statistically significant difference (U = 0, *p* = 0.025) in the mucosal epithelium (Table 8). In addition, no statistically significant differences in skin epithelium (U = 4.0, *p* = 0.054), blood vessels (U = 4.5, *p* = 0.120), sweat gland ducts (U = 1.0, *p* = 0.655), adipose glands (U = 0, *p* = 0.157), hair follicles (U = 1.0, *p* = 0.064), and connective tissue (U = 8.5, *p* = 0.381) were found (Table 8).

### 3.5. Appearance and Distribution of IL-6

**Figure 6:**
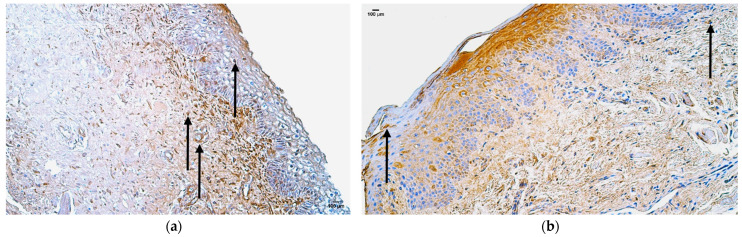
Immunohistochemistry of the IL-6-positive structures in the control and patient tissue samples: (**a**) control sample with few IL-6-positive structures in the skin epithelium and moderate in the blood vessels, sweat gland ducts, and connective tissue (arrows), 200×; (**b**) patient sample with few to moderate IL-6-positive structures in blood vessels, with moderate in skin epithelium and sweat gland ducts, and moderate to numerous in mucosal epithelium and connective tissue (arrows), 200×.

In the control group, the median quantity of IL-6-positive structures was few (+) in the skin epithelium and mucosal epithelium, moderate (++) in blood vessels, sweat gland ducts, and connective tissue, and moderate to numerous (++/+++) in adipose glands and hair follicles (Figure 6a, Table 7).

In the patient group, the median quantity of Gal-10-positive structures was few to moderate (+/++) in the blood vessels, moderate (++) in skin epithelium, hair follicles, and connective tissue, moderate to numerous (++/+++) in sweat gland ducts and adipose glands, and numerous to abundant (+++/++++) in the mucosal epithelium (Figure 6b, Table 7).

Comparison of both groups using the Mann–Whitney U test illustrates a statistically significant difference (U = 0, *p* = 0.031) in the mucosal epithelium (Table 8). In addition, no statistically significant differences in skin epithelium (U = 5.5, *p* = 0.119), blood vessels (U = 9.5, *p* = 0.515), sweat gland ducts (U = 1.0, *p* = 0.429), adipose glands (U = 2.0, *p* = 1.000), hair follicles (U = 4.5, *p* = 0.578), and connective tissue (U = 4.5, *p* = 0.059) were found (Table 8).

### 3.6. Appearance and Distribution of HBD-2

**Figure 7:**
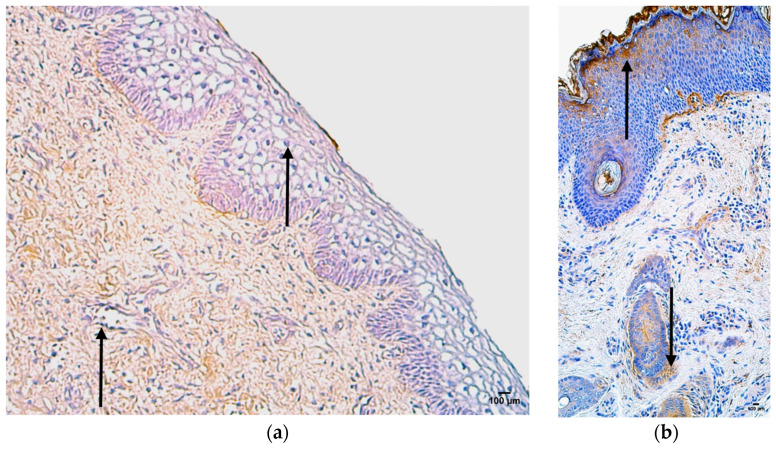
Immunohistochemistry of the HBD-2-positive structures in the control and patient tissue samples: (**a**) control sample with the absence of HBD-2-positive structures in skin epithelium, blood vessels, and connective tissue (arrows), 200×; (**b**) patient sample with rare occurrence of HBD-2-positive structures in connective tissue, moderate to numerous in skin epithelium and adipose glands, and numerous to abundant in mucosal epithelium (arrows), 200×.

In the control group, the median quantity of HBD-2-positive structures was none (0) in the skin epithelium, blood vessels, adipose glands, and connective tissue, and rare (0/+) in the mucosal epithelium, sweat gland ducts, and hair follicles (Figure 7a, Table 9).

In the patient group, the median quantity of HBD-2-positive structures was few (+) in the hair follicles, few to moderate (+/++) in skin epithelium, blood vessels, and sweat gland ducts, moderate (++) in adipose glands, and moderate to numerous (++/+++) in the mucosal epithelium (Figure 7b, Table 9).

Comparison of both groups using the Mann–Whitney U test illustrates statistically significant differences in skin epithelium (U = 0, *p* = 0.007), mucosal epithelium (U = 0, *p* = 0.032), blood vessels (U = 3.0, *p* = 0.034), and hair follicles (U = 0, *p* = 0.029) (Table 10). In addition, no statistically significant differences in the sweat gland ducts (U = 0, *p* = 0.057), adipose glands (U = 0, *p* = 0.057), and connective tissue (U = 0, *p* = 0.007) were found (Table 10).

### 3.7. Appearance and Distribution of HBD-3

**Figure 8:**
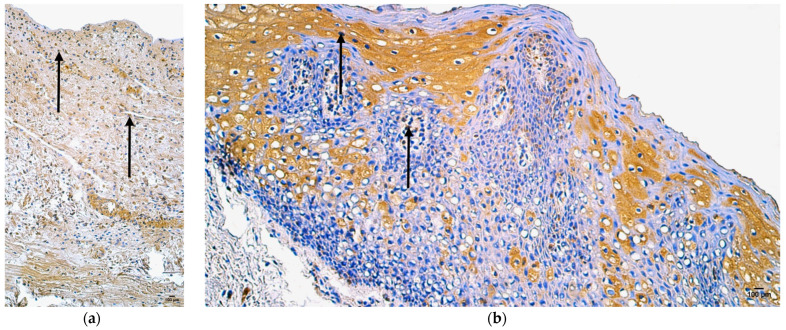
Immunohistochemistry of the HBD-3-positive structures in the control and patient tissue samples: (**a**) control sample with few HBD-3-positive structures in skin and mucosal epithelium, few to moderate in blood vessels, numerous in adipose glands, moderate to numerous in hair follicles, numerous in connective tissue (arrows), 200×; (**b**) patient sample with rare HBD-3-positive structures in connective tissue, few to moderate in blood vessels and skin epithelium, moderate in mucosal epithelium, numerous in adipose glands and hair follicles, numerous to abundant in sweat gland ducts (arrows), 200×.

In the control group, the median quantity of HBD-3-positive structures was few (+) in the skin epithelium, few to moderate (+/++) in the mucosal epithelium, blood vessels, and sweat gland ducts, moderate (++) in the adipose glands and connective tissue, and moderate to numerous (++/+++) in hair follicles (Figure 8a, Table 9).

In the patient group, the median quantity of HBD-3-positive structures was rare (0/+) in the connective tissue, few to moderate (+/++) in blood vessels, moderate (++) in skin epithelium and sweat glands, moderate to numerous (++/+++) in the mucosal epithelium and hair follicles, and numerous (+++) in adipose glands (Figure 8b, Table 9).

Comparison of both groups using the Mann–Whitney U test illustrates statistically significant differences in mucosal epithelium (U = 0, *p* = 0.019) and connective tissue (U = 1.5, *p* = 0.016) (Table 10). In addition, no statistically significant differences in the skin epithelium (U = 5.5, *p* = 0.135), blood vessels (U = 12.0, *p* = 0.914), sweat gland ducts (U = 2.0, *p* = 0.564), adipose glands (U = 3.0, *p* = 0.252), and hair follicles (U = 6.0, *p* = 1.000) were found (Table 10).

### 3.8. Appearance and Distribution of HBD-4

**Figure 9:**
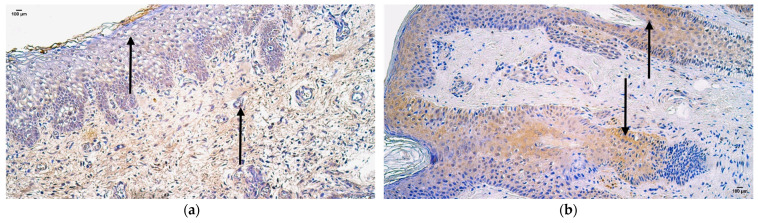
Immunohistochemistry of the HBD-4-positive structures in the control and patient tissue samples: (**a**) control sample with no HBD-4-positive structures in skin and mucosal epithelium, adipose glands, hair follicles, and connective tissue, rare in blood vessels (arrows), 200×; (**b**) patient sample with rare HBD-4-positive structures in blood vessels and connective tissue, moderate in skin epithelium, moderate to numerous in adipose glands and hair follicles, numerous in mucosal epithelium and sweat gland ducts (arrows), 200×.

In the control group, the median quantity of HBD-4-positive structures was rare (0/+) in the blood vessels and connective tissue, few to moderate (+/++) in sweat gland ducts, moderate (++) in skin epithelium and adipose glands, moderate to numerous (++/+++) in hair follicles, and numerous (+++) in the mucosal epithelium (Figure 9a, Table 11).

In the patient group, the median quantity of HBD-4-positive structures was none (0) in the skin epithelium, mucosal epithelium, sweat gland ducts, and connective tissue, and rare (0/+) in blood vessels, adipose glands, and hair follicles (Figure 9b, Table 11).

Comparison of both groups using the Mann–Whitney U test illustrates statistically significant differences in the skin epithelium (U = 0.5, *p* = 0.009), mucosal epithelium (U = 0, *p* = 0.015), and hair follicles (U = 0, *p* = 0.031) (Table 12). In addition, no statistically significant differences in blood vessels (U = 10.5, *p* = 0.606), sweat gland ducts (U = 0, *p* = 0.076), adipose glands (U = 0, *p* = 0.057), and connective tissue (U = 4.5, *p* = 0.065) were found (Table 12).

### 3.9. Appearance and Distribution of IL-10

**Figure 10:**
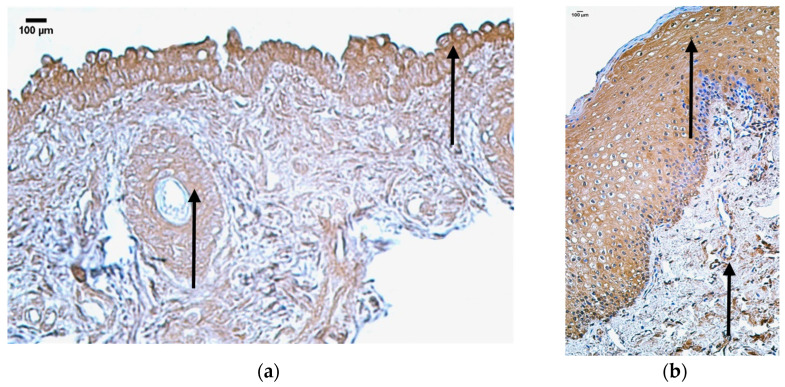
Immunohistochemistry of the IL-10-positive structures in the control and patient tissue samples: (**a**) control sample with rare occurrence of IL-10-positive structures in adipose glands, few in mucosal epithelium and blood vessels, moderate in skin epithelium, hair follicles, and connective tissue (arrows), 200×; (**b**) patient sample with few IL-10-positive structures in skin epithelium, few to moderate in connective tissue, moderate in blood vessels and hair follicles, numerous in mucosal epithelium and sweat gland ducts (arrows), 200×.

In the control group, the median quantity of CD-163-positive structures was rare (0/+) in the sweat gland ducts and adipose glands, few (0) in the mucosal epithelium, few to moderate (+/++) in blood vessels, moderate (++) in skin epithelium and hair follicles, and moderate to numerous (++/+++) in connective tissue (Figure 10a, Table 11).

In the patient group, the median quantity of IL-10-positive structures was few (+) in the skin epithelium and connective tissue, few to moderate (+/++) in adipose glands and hair follicles, moderate (++) in blood vessels, moderate to numerous (++/+++) in sweat gland ducts, and numerous (+++) in the mucosal epithelium (Figure 10b, Table 11).

Comparison of both groups with the Mann–Whitney U test illustrates statistically significant differences in the mucosal epithelium (U = 1.0, *p* = 0.037) and connective tissue (U = 0, *p* = 0.008) (Table 12). In addition, no statistically significant differences in skin epithelium (U = 12.0, *p* = 0.911), blood vessels (U = 10.5, *p* = 0.661), sweat gland ducts (U = 0, *p* = 0.180), adipose glands (U = 1.5, *p* = 0.374), and hair follicles (U = 4.5, *p* = 0.297) were found (Table 12).

### 3.10. Comparison of Defense Factor Appearance and Distribution

**Figure 11:**
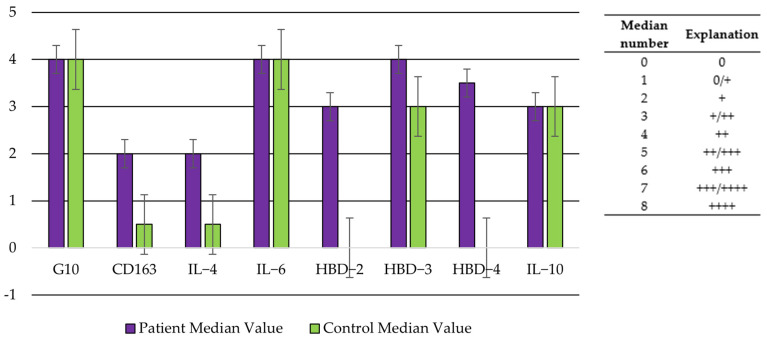
Comparison of immunity defense factor median distribution in patient and control group tissues.

A visual summary of the comparison between immunity defense factor median values in the patient group and control group tissue samples is illustrated in Figure 11, where the overall differences between CD163, IL4, and the HBDs’ distribution across the tissues can be seen. 

### 3.11. Correlation in the Epithelium and Structures of Connective Tissue of Patient Group

**Figure 12:**
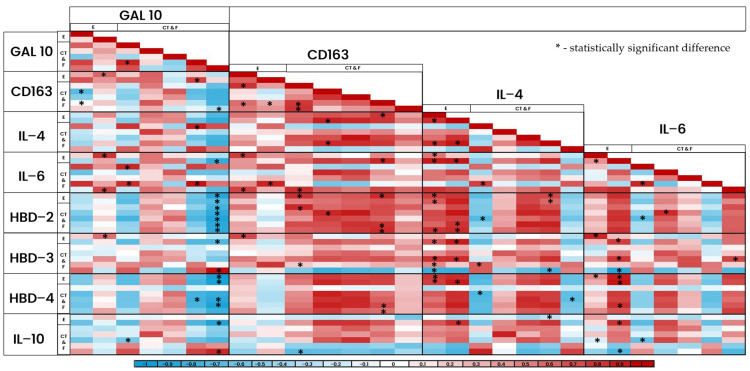
Heat-map of correlations between the factors (part one).

**Figure 13:**
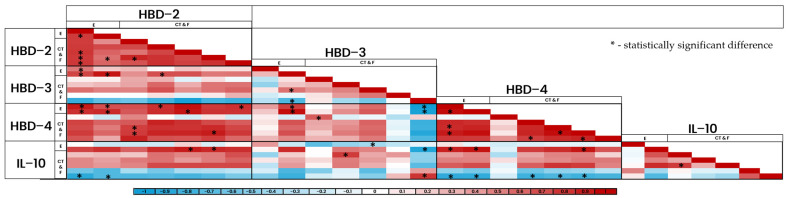
Heat-map of correlations between the factors (part two).

Using Spearman’s rank correlation coefficient, multiple statistically significant correlations (*p* < 0.005) were obtained between the factors in epithelium and connective tissue structures (Figure 12 and Figure 13). The factors presented both positive (red) and negative (blue) correlations, with the biggest amount of statistically notable correlations being present in the epithelium of the patient tissue samples.

## 4. Discussion

In our study, a statistically significant increase in HBD-2-, HBD-3-, and HBD-4-positive structures was found in skin and the mucosal epithelium, hair follicles, and blood vessels of patient samples. A notable increase was also seen in IL-4, IL-6, and IL-10 in the mucosal epithelium and CD163 in the blood vessels. The connective tissue of patient samples presented with statistically significant decreases in Gal-10, IL-10, and HBD-3.

CD-163 is known to be a specific and important marker for M2 anti-inflammatory macrophages and their alternative activation [11,13]. Due to the alternative activation path of M2 macrophages, they present as anti-inflammatory by increasing tissue repair, angiogenesis, and expressing anti-inflammatory markers and cytokines like IL-10 [13,60]. It is also well-known that macrophages, especially M2 macrophages, play a crucial role in the event of vascular injury by recovering blood flow, repairing the vessel, resolving inflammation, and promoting wound healing [61]. These angiogenetic functions are ensured by the enhancement of endothelial cell proliferation, migration, and interactions with PFGFBB, MMP, TGFB1, and VEGF [62]. In our study, CD-163 significantly increased around the blood vessels of the patient group; therefore, this leads to a possible suggestion that in bilateral cleft lip and palate, the M1/M2 macrophage equilibrium is possibly shifted toward M2 macrophages to ensure angiogenesis and, therefore, resolution of chronic inflammation and better tissue healing in order to regain tissue homeostasis.

Human beta-defensins are antimicrobial peptides that control the process of inflammation or infection by the elimination of pathogens, improvement of wound healing, as well as by controlling cell proliferation, migration, chemotaxis, and cytokine production [40,41,42,43]. They form the first line of defense of epithelial tissues that ensure immune response and homeostasis [38,39,44]. Changes in HBD levels suggest impaired functions of the epithelial and mucosal barrier and increased susceptibility of pathogen invasion and infection development [37,43,63]. They are most commonly secreted by epithelial cells, therefore, they are most prominent in the structure of the epithelium, as well as the salivary glands [37,40,64]. The main upregulating factor for HBD secretion is inflammation or infection; hence, the upregulation of human beta-defensins in skin epithelium, mucosal epithelium, hair follicles, and blood vessels indicate the presence of chronic inflammation, as well as the presence of a line of defense that tries to regain tissue homeostasis of cleft-affected tissue [37,38,39,44] and characterizes also our patient bilateral cleft-affected lips.

An increase in HBD-4 expression in the skin and mucosal epithelium and increase in HBD-3 expression in mucosal epithelium mostly indicates the antimicrobial effects via the formation of pores in the cell membranes of pathogenic microorganisms [37], and an increase in mast cell degranulation, chemotaxis, and cytokine production [41,48,49,50]. The increase in HBD-2 secretion in the skin and mucosal epithelium, blood vessels, and hair follicles as well as the increase in HBD-3 secretion suggests that the overexpression of these factors severely increases healing to overcompensate the damage of tissue that has been caused by the chronic inflammation and restore normal protective functions of the epithelial barrier [41]. Furthermore, in our study, a significant decrease in HBD-3 was observed in the connective tissue of the patient group, which suggests impaired protection against pathogens and increased susceptibility to infections [37,43,63]. The inversed expression of HBD-3 in connective tissue and mucosal epithelium can possibly be explained by the fact that due to the nature of the cleft lip defect, the factor is significantly more necessary in the epithelium where the defect is the most severe; therefore, the overexpression in the epithelium is balanced by under-expression in the underlying connective tissue.

IL-4 is an anti-inflammatory cytokine that reduces inflammation, maintains homeostasis, promotes tissue repair, and decreases the release of pro-inflammatory cytokines [15,17,19,65,66]. It is one of the first and most important modulators that start the Th2 immune response (allergy, parasites) by instigating the cascade of CD4+ T cell, Th2 cell, B cell, and plasma cell differentiation, IgE secretion, and macrophage accumulation [67,68]. In our study, IL-4 levels in the mucosal epithelium were increased, therefore, it can be suggested that IL-4 functions as a protective agent that prevents excessive damage of the tissue that can be caused by the organism’s immune response and the state of chronic inflammation [18]. Interestingly, when looking at functions of IL-4 in the epithelium and more specifically in keratinocytes, a healing-impairing function has been noted in a study conducted by Serezani et al. [69]. This effect could possibly be explained by the fact that with the decrease in healing properties in the mucosal epithelium, the overexpression of IL-4 ensures that the environment is suitable for the development of chronic infections.

IL-6 is a regulatory inflammatory cytokine that is secreted as a defense mechanism to tissue infections and injuries [23,24,25,26]. Increased levels of IL-6 promote active effects of the immune system via B- and T-cell differentiation, Ig secretion, ACP synthesis, and cell apoptosis [19,24]. However, decreased levels of IL-6 are important in the end stages of an immune reaction to ensure reparative functions and wound closure [70]. IL-6 has also been mentioned as an important promoter of chronic inflammation via regulation of T-cell differentiation [25,71]. Therefore, the increased expression of IL-6 in our study suggests that overexpression of IL-6 in the mucosal epithelium has a similar effect to the increase in IL-4—the factor promotes the state of inflammation and immune reaction, decreases the opportunity for the tissue to heal and, therefore, makes the tissue environment more suitable for chronic infections.

IL-10 also presents with anti-inflammatory effects by inhibiting pro-inflammatory cytokine secretion, antigen presentation, maintaining tissue homeostasis, and preventing tissue damage, all with the objective to resolve inflammation [28,30,72,73,74]. In Th1 cells, IL-10 ensures that the Th1 immune response is not excessive and destructive, and IL-10 from Th2 cells suppresses Th1 cells; however, in macrophages, it ensures the formation of a self-regulating macrophage population [28,31,75]. These functions that differ through cell types ensure that the immune response is regulated through all stages via different signaling pathways [29,30,76]. Due to IL-10′s anti-inflammatory nature, it is critical to carefully regulate and monitor its secretion to avoid excessive immunosuppression or immune activity [75]. In this study, the quantity of IL-10 was increased in mucosal epithelium, hence, the upregulation of this interleukin once again presents with the same effect and continues to add to the suppression of excessive inflammation and the creation of a favorable environment for prolonged infections [29,30]. Therefore, all three interleukins that were observed in this study create a high-functioning cascade that tries to increase healing and wound repair via different pathways; however, the effect that is created is more negative and makes the already infected and inflamed clefted tissue more suitable for the continuance of infections, immune responses, and tissue destruction.

Moreover, when looking at the connective tissue of the patient group, the IL-10 quantity was decreased. IL-10 is fundamental in the healing, wound repair, and scar formation process due to the fact that it increases epitheliocyte proliferation, hyaluronan synthesis, TGF- β expression, and induces ECM remodeling [73]. It has also been noted that IL-10 promotes the correct organization of collagen fibers during wound formation [35]. For these reasons, it can be suggested that the decrease in IL-10 observed in our study points to disrupted healing of the connective tissue and excessive scarring and fibrosis of the damaged area; therefore, making it more difficult for the clefted lip to heal properly and to be successfully repaired without compromising any functional or aesthetic parameters. 

Gal-10 is a protein mainly found in human eosinophils, which is released into the tissue during eosinophil extracellular trap cell death [5,6,77,78]. The main functions of this protein are connected to immune responses targeted to parasites or allergic substances, inflammatory cytokine, chemokine, and prostaglandin release, as well as to participate in the EETosis, which ensnares and kills the pathogenic microorganisms that have entered the body [5,6,8,77,79,80]. Based on this information, the decrease in Gal-10 in the connective tissue of the patient group points to decreased eosinophilic function which, therefore, makes the tissue more susceptible to pathogenic, allergic, and parasitic infections as well as eosinophilic inflammation [5,7,77]. In a study performed by Buschmann et al., the increased levels of Gal-10 were connected to the development and maintenance of chronic inflammation [5]. For this reason, the decrease observed in this study could also be explained as a protective mechanism, where the cells of the connective tissue are attempting to decrease the presence of inflammation, which is promoted by various other local tissue factors. It is also important to note that Gal-10 has been previously connected to various pathologies connected to the nasal region, therefore, the change in this factor in the case of bilateral cleft lip could also point to the pathophysiological connection between the lip and nose in the case of this orofacial defect.

When looking at statistically significant correlations, multiple positive and negative correlations were observed between all of the factors in various tissue locations—skin and mucosal epithelium, blood vessels, sweat and adipose glands, hair follicles, and connective tissue. Correlations do not always mean causality; therefore, the interpretation of these results cannot always indicate the formation of a meaningful link between the factors. However, the possible connections of the synergistic actions of these factors are described as follows:

Galectin-10:

1. CD-163. Several positive correlations were observed between Gal-10 and CD-163. Gal-10 is characterized by its eosinophilic functions, eosinophilic inflammation, and release during EETosis [5,6,78], mostly with a destructive nature. On the other hand, CD163 is connected to M2 macrophages, which are characterized by their anti-inflammatory nature, improvement of healing, and downregulation of inflammation [12,14]. In a study conducted by Liu et al., it was noted that in various bacterial and parasite infections, M2 macrophages protect the body from excessive tissue damage by clearing apoptotic cells and inducing regeneration [81]; hence, connecting the functions of these two factors, it can be suggested that this positive correlation between them indicates mutual synergy, where Gal-10 creates tissue inflammation to protect it from invaders but M2 macrophages work alongside it to clear the created debris and to preserve the tissue from extreme inflammatory damage;
2. IL-6. Mostly positive correlations in the epithelial parts of the samples, which point to a mutual increase in inflammatory reactions;
3. IL-4. One positive correlation between IL-4 and Gal-10 was observed, which can be explained by the similar secretion mechanisms of both factors—either can be secreted by eosionphils, basophils, or T cells [5,6,15,16,17,67,68,77];
4. HBD-2, -3, and -4. Mostly negative correlations and only in the connective tissue were observed between the human beta-defensins and galectin-10. This could mean that the increased activity of Gal-10 and eosinophilic functions suppress the antimicrobial and healing properties of human beta-defensins, creating an environment more suitable for developing and sustaining chronic inflammation;
5. IL-10. Similarly, correlations dominate in the connective tissue and are mostly negative, which continues the link of increased inflammation via Gal-10 activation and the suppression of healing and restorative properties through a decrease in anti-inflammatory IL-10.

CD-163:

1. IL-4. In order for macrophages to polarize into the M2 subtype, they have to go through an alternative activation path during the inflammatory response with IL-1, IL-4, IL-6, IL-13, TGF-β, TLR, and glucocorticoids [11,13]. Polarization of the macrophages into M1 (killer) or M2 (healer) phenotypes creates a delicate equilibrium between pro- and anti-inflammatory occurrences in the body and ensures balance between inflammation and its resolution [60,82]. Some positive correlations were noted between IL-4 and CD-163 in the epithelium and connective tissue, which can be explained by the fact that macrophage polarization into the M2 subtype—which expresses CD-163—is carried out through an alternative activation path during the inflammatory response with IL-4 as a mediating agent [11,13]. Previously, possible connections between IL-4 and M2 macrophages and tissue healing have been suggested [83];
2. IL-6. Similarly, positive correlations were observed with IL-6 because IL-6 also increases M2 macrophage polarization and CD-163 expression [11,12,13,84]. Moreover, M2 macrophages have been noted to induce IL-6 secretion [12,14];
3. IL-10. One negative correlation in the connective tissue was seen. This finding is contrary to previous studies, where IL-10 was noted to induce M2 polarization [85] and where M2 macrophages secreted IL-10 [12,14]. IL-10 has also been noted to maintain tissue homeostasis and resolve inflammation with inhibiting antigen presentation to macrophages [28,30,72,73,74]. Therefore, the finding of our study could point to the suppression of IL-10 with the aim of maintaining antigen presentation and active functions of M2 macrophages;
4. HBD-2, -3, and -4. Several positive correlations were noted, which can be explained by the similar functions of these factors—all of them can improve healing, angiogenesis, antimicrobial effects, and inflammation resolution [12,14,40,41,42,43].

IL-4:

1. IL-6. Several positive correlations were noted between the factors. In previous studies, in order for the tissue and wound to heal properly and without any complications, it had been noted that IL-6 and IL-4 levels change inversely—during the healing stages IL-6 decreases but IL-4 increases to facilitate wound repair and M2 activity [70,86,87]. Therefore, the results of this study are contrary as they suggest that in the case of cleft lip, the mechanism of normal wound healing is impaired and the patient is more susceptible to infections, fibrosis, scarring, and hypertrophic scar formation [70];
2. IL-10. One positive correlation in the epithelium was observed between IL-4 and L-10. Both of these factors are mentioned to promote tissue repair, reduce inflammation, maintain homeostasis, and suppress excessive inflammation [28]. Moreover, a possible link between these factors has been previously noted as well as the fact that IL-10 can be upregulated by IL-4 [28,33,83]. For these reasons, it is clear that IL-10 and IL-4 both function synergistically to fight the extensive inflammation that is characteristic of cleft-affected tissue;
3. HBD-2, -3, and -4. A vast amount of positive correlations were observed between HBD-2, -3, and -4 and some negative correlations were also present between IL-4 and HBD-4. The positive correlations can also be explained by the similar anti-inflammatory functions of the factors but the negative correlation between IL-4 and HBD-4 could suggest impairment of the epithelial barrier and its protective functions [48].

IL-6:

1. IL-10. Mostly negative correlations were observed, which are in accordance with previous studies—where, during the tissue-healing phase, IL-6 decreases and IL-10 increases to facilitate successful wound repair [86,87]. However, some studies have also shown directly increasing links between IL-6 and IL-10, mostly in cases of chronically driven inflammation where prominent tissue degeneration is present [27,86,87,88,89]. Therefore, it can be assumed that in the tissues of cleft lip, IL-6 and IL-10 are cooperating in a manner of maximal healing induction to promote faster and more adequate wound repair;
2. HBD-2, -3, and -4. Mostly positive correlations were observed between IL-6 and all of the human beta-defensins, which suggest that human beta-defensins and IL-6 mutually stimulate one another to facilitate and drive the inflammation toward the desired resolution of the process [39,40,41,48,49,50].

HBDs:

3. IL-10. Both positive and negative correlations were observed between IL-10 and HBD-2, -3, and -4. The positive correlations can be explained by the anti-inflammatory mutual stimulation and induction of the factors to ensure more prominent healing, repairing, and homeostatic effects [39,40]. In addition, the negative correlations could point to IL-10′s downregulating effects on the HBD’s pro-inflammatory effects, like antimicrobial action and induction of immune and inflammatory reactions [90];
4. HBDs. All of the human beta-defensins were mutually positively correlated in the epithelium and connective tissues of the samples, which continues to suggest the similar functions of all these antimicrobial peptides and their link with one another in order to induce the most effective and comprehensive protective effects for the cleft-affected lip tissue. However, there was also a negative correlation between HBD-3 and HBD-4, which is contrary to previous studies—where a strong link between HBD-3 and HBD-4 and their functions has been described [37,39]. This leads to believe that the cooperative effect between these factors that usually ensures comprehensive protection of epithelium, mucosa, and the underlying tissue is severely impaired in the case of cleft lip.

Moreover, when comparing the results of this study with results obtained in previous studies researching the same factors in cleft palate-affected patients of the same age group, it can be seen that in the case of the cleft lip, local tissue defense factors present with more notable increases in their levels but in cleft palate they were mostly decreased [91]. This phenomenon can be explained by the fact that cleft lip is a defect that is morphologically and clinically more connected to the outer environment; therefore, it should present with more prominent inflammatory and immunological processes. The deviance that was consistent in both of these studies is the decrease in HBD-3 in connective tissue, which continues to prove the significance of HBD-3 in cleft defects and its role in the suppression of connective tissue healing and homeostasis. In both studies, the correlations between HBDs were also similar, which continues to be a reliable proof of the changes in the epithelial barriers and healing properties of cleft-affected tissues.

It is also noteworthy to mention that the connections between clefted tissue and local tissue defense factors observed in this study are novel findings due to the severity of the defect that is present in patients with bilateral cleft lip. Bilateral cleft lip is the most severe form of cleft, therefore, the patient presents with a distinct scarcity of tissue material. This results in not only more technically difficult surgery but also in almost impossible acquisition of excess tissue material for scientific samples. Moreover, the novelty of this study is further extended with the young age of the patients and scarcity of other studies researching this specific age group (until the age of milk dentition). 

A noteworthy limitation of our study could be the inconsistent presence of all tissue structures in the patient tissue samples—some lacked hair follicles, adipose glands, sweat glands, or some parts of the epithelium. This limitation is closely connected to the severity of the bilateral cleft lip defect and scarcity of the tissue material present. The main causal factor for this limitation is the fact that the tissue samples can only be acquired when they are not needed for the sufficient repair of the clefted defect during cheiloplasty surgery. Due to the reason that the patients are young and small, the amount of tissue necessary for the reconstruction is already scarce, so any additional and unneeded tissue material is rarely present in these cases. Moreover, the tissue pieces that can be used for scientific sample acquisition will not always be identical and with the same tissue structures present because all of the best parts of the tissue have been used for the patient reconstruction, and every case of BCLP and its reconstruction is different and requires different approaches to close the defect with only using the available tissue present. For this reason, the tissue that is left will always be different and sometimes may even be damaged during the reconstructive process, therefore, missing certain tissue structures.

Moreover, to gain a clearer insight about the defense factor concentration and gene distribution, other methods like ELISA and in situ hybridization could be used as well as longitudinal correlations between the changes in the factors and clinical state of the patients throughout the years and many follow-up surgeries.

## 5. Conclusions

Generally, in BCLP tissues, an increase in HBDs, M2 macrophages, IL-4, and IL-6, was observed, while IL-10 and Gal-10 showed a significant decrease.

The increase in ILs and HBDs suggests increased healing and wound repair, which is complemented by chronic inflammation and a larger susceptibility to chronic and continuous infections.

An attempt to reduce the presence of inflammation could be explained by the decrease in IL-10 and Gal-10, which points to compromised and fibrotic tissue healing as well as decreased eosinophilic functions.

The increase in CD163 around the blood vessels could signal a possible shift in the M1/M2 macrophage equilibrium toward M2 macrophages to ensure angiogenesis, resolution of inflammation, and tissue healing.

The presence of various positive and negative mutual correlations between the factors indicates mutually linked effects, especially between HBDs and ILs, which function as important and synergistic mediators of immunological, inflammatory, and homeostatic processes that occur in the tissues of bilateral cleft lip.

## Figures and Tables

**Table 1:** Description of the patients.

Patient Number	Age (Months)	Sex	Remarks
238	4	M	
264	4	F	
396	4	F	Mother did not use medication during pregnancy
395	6	F	Mother with a cleft
369	17	F	

Abbreviations: M—male; F—female.

**Table 2:** Description of the control group.

Control Number	Age	Sex	Cause of Death
1a	Newborn	M	Birth asphyxia
2a	Newborn	F	Birth asphyxia
4a	24 weeks old	F	Abortion due to the maternal indication
5a	Newborn	F	Sudden infant death syndrome
6a	Newborn	F	Sudden infant death syndrome

Abbreviations: M—male; F—female.

**Table 3:** Information about the antibodies used in IHC.

Tissue Factor	Product Code	Working Dilution	Company	Location
Gal-10	ab157475	1:200	Abcam	Cambridge, UK
CD 163	ab87099	1:200	Abcam	Cambridge, UK
IL-4	orb10908	1:100	Biorbyt	Cambridge, UK
IL-6	sc-28343	1:100	Santa Cruz Biotechnology Inc.	Santa Cruz, CA, USA
IL-10	orb100193	1:600	Biorbyt LLC	St Louis, MO, USA
HBD-2	sc-20798	1:100	Santa Cruz Biotechnology Inc.	Dallas, TX, USA
HBD-3	orb183268	1:100	Biorbyt LLC	St Louis, MO, USA
HBD-4	ab70215	1:100	Abcam	Cambridge, UK

**Table 4:** Explanation of semi-quantitative evaluation identifiers [56,57].

Identifier Used	Explanation
0	No positive structures (0%)
0/+	Rare occurrence of positive structures (12.5%)
+	Few positive structures (25%)
+/++	Few to moderate number of positive structures (37.5%)
++	Moderate number of positive structures (50%)
++/+++	Moderate to numerous positive structures (62.5%)
+++	Numerous positive structures (75%)
+++/++++	Numerous to abundant positive structures (87.5%)
++++	Abundance of positive structures (100%)

**Table 5:** Semi-quantitative evaluation of Gal-10 and CD-163 in control and patient groups.

Sample Number	Gal-10							CD-163						
	SE	ME	BV	SG	AG	F	CT	SE	ME	BV	SG	AG	F	CT
396	+/++	++/+++	+	++	+++	+	0	0	0	+	+++	++	0/+	++
369	++/+++	-	++	++	++	++	0/+	+	0	+/++	+	+/++	0/+	++
395	++/+++	+++	+/++	-	-	-	0	0/+	0/+	+	+	-	+	+
264	++	+++	0/+	-	-	-	0	+	0/+	++/+++	+	-	-	++/+++
238	++/+++	+++	+++	+++	-	+++	0	++	+/++	++	++	-	++	++
Patient Median	++/+++	+++	+/++	++	++/+++	++	0	+	0/+	+/++	+	++	+	++
1a	+	+	++	-	++/+++	+++	++	0	0	0/+	-	-	0	+
2a	++++	+	++	-	++	+++	++	0	0/+	+	0/+	-	-	++
4a	+++/++++	+++/++++	+++	-	-	++++	++/+++	+	+/++	+	+	-	-	++
5a	++/+++	++		++	+++	-	++	0	-	+	+	-	-	++
6a	++	+++	++	++	+++	++	++	0	0	0	-	0	0	0
Control Median	++/+++	++	++	++	+++	++	++	0	0/+	+	+	0	0	++

Abbreviations: SE—skin epithelium; ME—mucosal epithelium; BV—blood vessels; SG—salivary glands; AG—adipose glands; F—hair follicles; CT—connective tissue; Gal-10—galectin-10.

**Table 6:** Median values for Gal-10 and CD-163 in patient and control groups.

	Gal-10							CD-163						
	SE	ME	BV	SG	AG	F	CT	SE	ME	BV	SG	AG	F	CT
P	++/+++	+++	+/++	++	++/+++	++	0	+	0/+	+/++	+	++	+	++
C	++/+++	++	++	++	+++	++	++	0	0/+	+	+	0	0	++
U-test value	10.0	6.5	5.0	2.0	3.5	2.5	0.0	5.0	9.5	3.0	3.0	0.0	0.0	8.0
*p*-value	0.589	0.368	0.199	0.414	0.803	0.195	0.005	0.090	0.896	0.034	0.124	0.221	0.057	0.288

Abbreviations: SE—skin epithelium; ME—mucosal epithelium; BV—blood vessels; SG—salivary glands; AG—adipose glands; F—hair follicles; CT—connective tissue; Gal-10—galectin-10.

**Table 7:** Semi-quantitative evaluation of IL-4 and IL-6 in control and patient groups.

Sample Number	IL-4							IL-6						
	SE	ME	BV	SG	AG	F	CT	SE	ME	BV	SG	AG	F	CT
396	+	+++	0	++	0/+	+	0	+	+++	+	+++	+++	+/++	++
369	0	-	0	+	0/+	0/+	0	++	-	++	++	++	++	++
395	++	++/+++	-	-	-	-	0	+++	+++/++++	0/+	-	-	-	++
264	+	++/+++	0	-	-	-	+	++	++/+++	+/++	++	-	-	++/+++
238	++	+++	0/+	++/+++	-	++/+++	+	++/+++	+++/++++	++/+++	++/+++	-	+++	++/+++
Patient Median	+	+++	0	++	0/+	+	0	++	+++/++++	+/++	++/+++	++/+++	++	++
1a	0	0/+	-	-	0/+	+/++	0	+	+	0/+	-	-	++	+
2a	0	0/+	-	-	0	0/+	0	+	+	++	-	-	++/+++	+/++
4a	-	0/+	-	-	0/+	0/+	-	++	-	++/+++	-	+++	+++	++
5a	-	0/+	+/++	-	-	+	-	+	-	++	++	-	-	++
6a	0	0	-	0	0	0	0	++	++	++	-	++	++	++
Control Median	0	0/+	+/++	0	0/+	0/+	0	+	+	++	++	++/+++	++/+++	++

Abbreviations: SE—skin epithelium; ME—mucosal epithelium; BV—blood vessels; SG—salivary glands; AG—adipose glands; F—hair follicles; CT—connective tissue; IL-4—interleukin-4; IL-6—interleukin-6.

**Table 8:** Median values for IL-4 and IL-6 in patient and control groups.

	IL-4							IL-6						
	SE	ME	BV	SG	AG	F	CT	SE	ME	BV	SG	AG	F	CT
P	+	+++	0	++	0/+	+	0	++	+++/++++	+/++	++/+++	++/+++	++	++
C	0	0/+	+/++	0	0/+	0/+	0	+	+	++	++	++/+++	++/+++	++
U-test value	4.0	0.0	4.5	1.0	0.0	1.0	8.5	5.5	0.0	9.5	1.0	2.0	4.5	4.5
*p*-value	0.054	0.025	0.120	0.655	0.157	0.064	0.381	0.119	0.031	0.515	0.429	1.000	0.578	0.059

Abbreviations: SE—skin epithelium; ME—mucosal epithelium; BV—blood vessels; SG—salivary glands; AG—adipose glands; F—hair follicles; CT—connective tissue; IL-4—interleukin-4; IL-6—interleukin-6.

**Table 9:** Semi-quantitative evaluation of HBD-2 and HBD-3 in control and patient groups.

Sample Number	HBD-2							HBD-3						
	SE	ME	BV	SG	AG	F	CT	SE	ME	BV	SG	AG	F	CT
396	+/++	+++	+	++/+++	++	+	0/+	0/+	+++	++	++	+++	++/+++	0/+
369	+/++	-	+/++	+	0/+	+	0	++	-	++	-	++	++	++
395	++/+++	+++/++++	0	-	++/+++	-	0/+	+++	+++/++++	0	-	-	-	0
264	++/+++	++	+/++	+	++	-	0/+	++	++	+	0/+	-	-	0/+
238	+/++	+	++	++	-	++	+/++	+/++	++	+/++	+++/++++	+++	+++	0/+
Patient Median	+/++	++/+++	+/++	+/++	++	+	0/+	++	++/+++	+/++	++	+++	++/+++	0/+
1a	0	0/+	0	-	-	0/+	0	+	+	+	-	+/++	++/+++	++
2a	0/+	0/+	0/+	-	0	0/+	0/+	+	+	+/++	-	++	++/+++	+++
4a	0/+	-	0	-	0	0	0	+/++	+/++	++	-	+++	+++	++/+++
5a	0	-	0	0/+	-	-	0	0/+	-	+	+/++	-	-	++
6a	0	0	0	0/+	-	0	0	+/++	+/++	+/++	+	++	++	++
Control Median	0	0/+	0	0/+	0	0/+	0	+	+/++	+/++	+/++	++	++/+++	++

Abbreviations: SE—skin epithelium; ME—mucosal epithelium; BV—blood vessels; SG—salivary glands; AG—adipose glands; F—hair follicles; CT—connective tissue; HBD-2—human beta-defensin 2; HBD-3—human beta-defensin 3.

**Table 10:** Median values for HBD-2 and HBD-3 in patient and control groups.

	HBD-2							HBD-3						
	SE	ME	BV	SG	AG	F	CT	SE	ME	BV	SG	AG	F	CT
P	+/++	++/+++	+/++	+/++	++	+	0/+	++	++/+++	+/++	++	+++	++/+++	0/+
C	0	0/+	0	0/+	0	0/+	0	+	+/++	+/++	+/++	++	++/+++	++
U-test value	0.0	0.0	3.0	0.0	0.0	0.0	0.0	5.5	0.0	12.0	2.0	3.0	6.0	1.5
*p*-value	0.007	0.032	0.034	0.057	0.057	0.029	0.007	0.135	0.019	0.914	0.564	0.252	1.000	0.016

Abbreviations: SE—skin epithelium; ME—mucosal epithelium; BV—blood vessels; SG—salivary glands; AG—adipose glands; F—hair follicles; CT—connective tissue; HBD-2—human beta-defensin 2; HBD-3—human beta-defensin 3.

**Table 11:** Semi-quantitative evaluation of HBD-4 and IL-10 in control and patient groups.

Sample Number	HBD-4							IL-10						
	SE	ME	BV	SG	AG	F	CT	SE	ME	BV	SG	AG	F	CT
396	++	+++	0/+	+++	++/+++	++/+++	0/+	+	+++	++	++/+++	++/+++	0/+	+
369	+	-	++	+/++	+	++	0/+	+	-	+	++	0/+	+/++	0/+
395	++/+++	+++	0	-	-	-	0	++/+++	+++	++	-	-	-	+
264	++	++	0/+	0/+	-	-	0/+	++	+/++	0/+	-	-	+	+
238	++	+++	0/+	-	-	++/+++	+	+	+++	++	+++	-	++	+/++
Patient Median	++	+++	0/+	+/++	++	++/+++	0/+	+	+++	++	++/+++	+/++	+/++	+
1a	0	0	0/+	-	0	0/+	0	++	++	+/++	-	-	+++	+++
2a	0	0	0/+	0	0/+	0/+	0	++	+	++	-	++	++	+++
4a	0	0	0/+	-	0	0	0	+	0/+	+	-	0	0/+	++/+++
5a	0	-	0	0	-	-	0	0/+	-	+/++	0/+	-	-	++/+++
6a	+	0/+	0/+	-	0/+	+	0/+	++	+	+	-	0/+	++	++
Control Median	0	0	0/+	0	0/+	0/+	0	++	+	+/++	0/+	0/+	++	++/+++

Abbreviations: SE—skin epithelium; ME—mucosal epithelium; BV—blood vessels; SG—salivary glands; AG—adipose glands; F—hair follicles; CT—connective tissue; HBD-4—human beta-defensin 4; IL-10—interleukin-10.

**Table 12:** Median values for HBD-4 and IL-10 in patient and control groups.

	HBD-4							IL-10						
	SE	ME	BV	SG	AG	F	CT	SE	ME	BV	SG	AG	F	CT
P	++	+++	0/+	+/++	++	++/+++	0/+	+	+++	++	++/+++	+/++	+/++	+
C	0	0	0/+	0	0/+	0/+	0	++	+	+/++	0/+	0/+	++	++/+++
U-test value	0.5	0.0	10.5	0.0	0.0	0.0	4.5	12.0	1.0	10.5	0.0	1.5	4.5	0.0
*p*-value	0.009	0.015	0.606	0.076	0.057	0.031	0.065	0.911	0.037	0.661	0.180	0.374	0.297	0.008

Abbreviations: SE—skin epithelium; ME—mucosal epithelium; BV—blood vessels; SG—salivary glands; AG—adipose glands; F—hair follicles; CT—connective tissue; HBD-4—human beta-defensin 4; IL-10—interleukin-10.

## Data Availability

All datasets used in the present study are available in Section 3.
